# Prevalence and Associated Risk Factors of Suicidal Ideation Among Brazilian Pregnant Women: A Population-Based Study

**DOI:** 10.3389/fpsyt.2022.779518

**Published:** 2022-03-22

**Authors:** Alexandre Faisal-Cury, Daniel Maurício Oliveira Rodrigues, Alicia Matijasevich, Fernanda Tarpinian, Karen Tabb

**Affiliations:** ^1^Departamento de Medicina Preventiva da, Faculdade de Medicina FMUSP, Universidade de São Paulo, Brazil; ^2^Faculdade de Medicina de Jundiaí, Jundiaí, São Paulo, São Paulo, Brazil; ^3^University of Illinois at Urbana-Champaign, Champaign, IL, United States

**Keywords:** antenatal depression, minority ethnic women, prenatal care, screening, suicidal ideation

## Abstract

**Background:**

Suicide is a leading cause of death during the perinatal period in high-income countries (HIC). There remains a lack of population-based studies about suicidal ideation (SI) during pregnancy among low and middle income countries (LMIC).

**Objective(s):**

Using the case of Brazil, we aim to estimate the prevalence of SI during pregnancy and its association with antenatal depression (AD) and sociodemographic factors in a LMIC.

**Method:**

We used data from the Brazilian National Survey (PNS-2019), a population-based study, with a complex and probabilistic sampling method. Of the 27,136 women of reproductive age (15 to 49 years old) who participated in the PNS, a total of 769 women reported being pregnant at the time of the interview. All PNS participants answered the Patient Health Questionnaire-9 (PHQ-9) and a questionnaire with sociodemographic data. SI was defined as any answer to the PHQ-9 item 9 other than 0 (not at all). Logistic regression models were performed to obtain crude and adjusted odds ratios (aOR) and 95% confidence intervals (95% CI) for the association between explanatory variables and SI during pregnancy.

**Results:**

Among 769 women, 33 (3.9%, 95% CI: 3.0–5.1%) reported SI during pregnancy. In the adjusted analysis, higher odds of SI were associated with being 20 to 34 years old (aOR:0.24, 95% CI: 0.08–0.74) or 35 to 49 years old (aOR:0.15; 95% CI: 0.04–0.50), having 9 to 11 years of education (aOR 0.23, 95% CI: 0.61–0.86), acheiving the highest family income category (aOR:0.08, 95% CI: 0.01–0.58), not living in the South/Southeast regions of Brazil (aOR:5.52, 95% CI: 2.36–12.9), and having probable mild AD (aOR:10.5 95% CI: 2.3–47.9) or moderate AD (aOR:241.3, 95% CI: 58.4–996.7).

**Conclusion(s):**

In Brazil, SI affects almost 4% of pregnant women and is associated with sociodemographic vulnerability. Clinically, women with mild symptoms of depression may also experience SI during pregnancy. These findings are important for designing effective perinatal mental health interventions in LMICs.

## Introduction

Suicide is a leading cause of death during the perinatal period worldwide, and there is much variation among countries (1). In high-income countries suicide accounts for 5% to 20% of maternal deaths (1). In comparison, among low- and middle-income countries the pooled prevalence of pregnancy-related deaths attributed to suicide is 1.7% (2). Suicidal ideation (SI), often a precursor to suicide, commonly called suicidal ideas or thoughts, is a broad term used to describe non-active suicidality including preoccupations, contemplations, or wishes of death. Sometimes SI, combined with suicide plans, attempts, or self-harming such as cutting, is assessed as suicidal behavior. Nevertheless, there is no universally accepted definition of SI, which creates an increased challenge for health providers and researchers. SI with high intent is an important distal predictor of later suicide death (3). A review of SI in people in 17 countries reported that 60% of transitions, from ideation to planning and attempting suicide, occur within the first year after SI onset (4). Another study of suicide, conducted in the US, also found rapid transition from onset of ideation to planning or attempting suicide (5). Accordingly, suicidal ideation is an important and often overlooked possible antecedent for mortality among high-risk populations such as pregnant people.

The reported prevalence of antenatal SI or thoughts has varied widely between studies depending on the SI definition, methods of evaluation, and sample characteristics, and ranges from 5 to 14% of women surveyed (6). In particular, pregnant adolescents (7, 8) and patients with psychiatric disorders are at greater risk of SI during pregnancy (6). A review of 17 studies in high- and low-income countries found a SI prevalence among pregnant and postpartum women ranges from 5 to 18% (9). Studies performed in LMICs such as India and South Africa found a SI prevalence of 7.6 and 18%, respectively (10, 11). There are several risk factors for SI during pregnancy. The characteristics of SI can be individual such as having personal and/or family history of psychiatric disorders and/or history of suicidal attempts, being unmarried, beign of younger age, or experiencing intimate partner violence (11–13). The characteristics of SI can also be related to socioeconomic situations such as living in poverty, having a lack of social support, experiencing social and/or racial inequalities, living in a rural area, having lower educational attainment (11–13). Lastly, characteristics of SI can be obstetric related factors such as unplanned pregnancy, nulliparity, or experiencing a complicated delivery (12–14). Examining risk factors that can be addressed or treated is an important step for suicide prevention.

Depression is one of the most important ameriolable risk factors for SI that can be treated if detected early (5, 9, 15, 16). Antenatal depressive (AD) symptoms have been associated with a 13- to 17-fold increase in odds of SI even after adjusting for covariates (15, 17). Nevertheless, not all women with SI report elevated depressive symptoms. For example, Zhong et al. (18) ADDIN EN.CITE (18) evaluated SI among 1,519 pregnant women attending prenatal care clinics in Lima, Peru, and found that 49% of participants who screened positive for SI screened negative for depressive symptoms. Moreover, participants with low scores for probable depression according to the Patient Health Questionnaire (PHQ-9) still endorsed SI. Despite the fact that depressive symptoms are highly associated with SI, only a portion of pregnant women presenting SI experience the co-occurrence of depression. Garman et al. (19) assessed depressive symptoms and suicidality among 384 pregnant women in South Africa and concluded that depression and suicide are overlapping but independent phenomena, especially among older and more chronically depressed perinatal women. These findings highlight the importance of paying special attention to SI during pregnancy and suggest that SI screening should be recommended for pregnant women both with and without depression (9). The frequent contact between pregnant women and providers makes prenatal appointments a unique opportunity for the diagnosis and intervention of this serious public health problem. The primary aim of the present study was to estimate the prevalence of SI among a population-based sample of pregnant women in Brazil, and the secondary aim was to assess any association between AD and sociodemographic risk factors with SI during pregnancy.

## Materials and Methods

### Design and Sample

**Figure 1:**
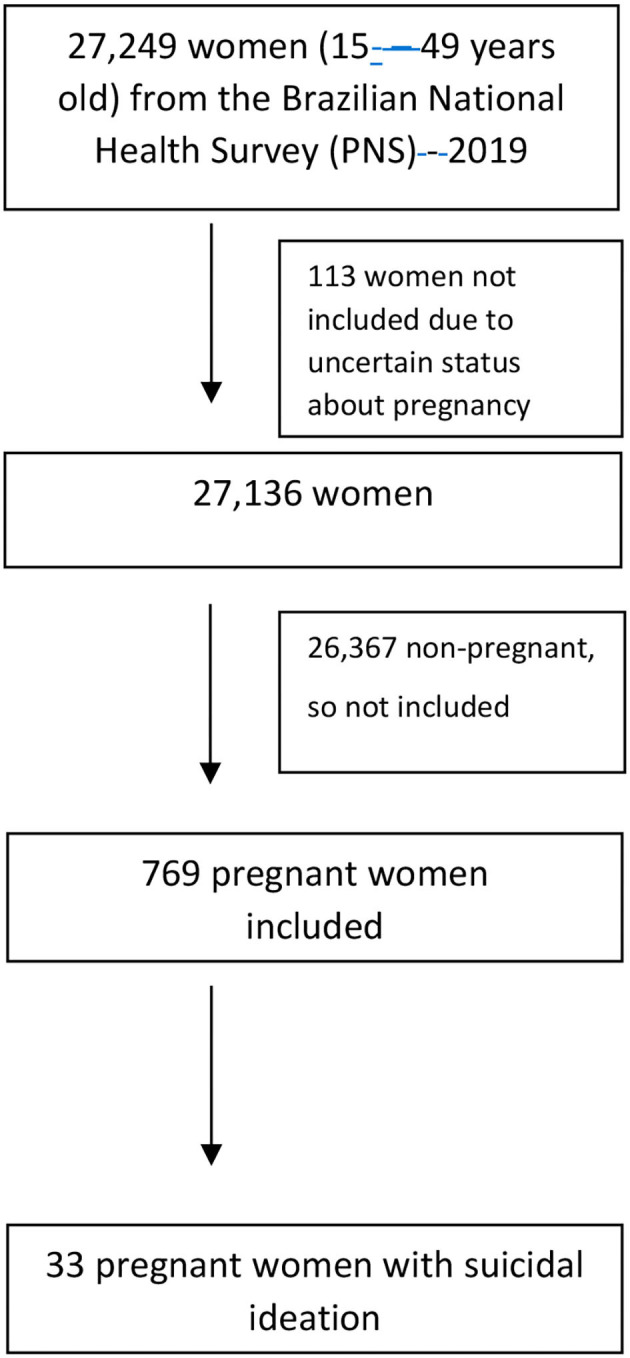
Participant flowchart.

The present study is a secondary analysis from the Brazilian National Health Survey (PNS- 2019), which was carried out from August 2019 to March 2020. Authors from the current study were not involved in the original data creation. PNS-2019 uses a multi-stage clustered sample. The expected sample size was 108,255 households, considering a 20% non-response rate, this number guaranteed a statistical power of 80%, and thus a sufficient estimate of health indicators. Data collection was performed through interviews conducted by trained field workers using a questionnaire format with a mobile data collection device. Further details of PNS-2019 are available elsewhere (20). The questionnaire solicited three types of information, which covered (i) characteristics of the household; (ii) all residents of the household, focusing on socioeconomic and health information; and (iii) the selected resident for whom lifestyle, chronic disease, violence, sexual behavior, and infectious diseases were investigated. For our analyses, we selected 27,249 women of reproductive age (15–49 years old) with 769 reporting being pregnant at the time of the interview (Figure 1).

### Main Outcome (Suicidal Ideation)

Suicidal ideation during pregnancy was assessed using item 9 from the Patient Health Questionnaire (PHQ-9). The PHQ-9 is a version of the PRIME-MD diagnostic instrument for common mental disorders (namely depression and anxiety). The PHQ-9 is the depression module, which scores each of the 9 DSM-IV criteria (20). The presence and intensity of each of the 9 items in the 2 weeks preceding the interview was determined. PNS-2019 did not provide information about trimesters or gestational period. The scores range from 0 (“not once”) to 3 (“almost every day”), and the total score can range from 0 to 27. Regarding PHQ-9 item 9 ("Thoughts that you would be better off dead, or of hurting yourself in some way”), there are four possible answers: 0 (not at all); 1 (several days); 2 (more than half the days); and 3 (nearly every day). Suicidal ideation was defined exclusively as any answer other than 0 (not at all). The PHQ-9 was validated in Brazil using a structured interview based on the DSM-IV (the gold standard) and can differentiate between probable cases and non-cases of depression (21). The specificity of the PHQ-9 suicide screening item was found to be 0.84 and the sensitivity 0.69 in a previous study (22). The reliability coefficient, Cronbach's alpha, for the PHQ-9 total score was 0.84 (23). The PHQ-9 was also used for classification of AD. We used three categories of probable depression: no depression (score <5), mild depression (score 5–9), and moderate depression (score >9). A PHQ-9 score >9 had a sensitivity of 88 and a specificity of 88% for major depression (24).

### Covariates

On the basis of the PNS-2019 questionnaire data, the following sociodemographic factors were assessed: participant's age (15–19, 20–34, 35–49 years of age); self-reported skin color (non-White or White); partner status (yes or no); place of residence (urban or rural); schooling in years (0–8, 9–11, >11); country region according to economic development (more developed: South/Southeast or less developed: North/Northeast/Midwest); having a private medical plan (no or yes); and family monthly per capita income according to the Brazilian minimum wage ($1 minimum wage = $USD242.20) (0-½; ½-1; >1). Family monthly income per capita was estimated by dividing family income by the number of persons living in the household. Self-perception of health status was also collected and was classified as very good/good, regular, or poor/very poor.

### Statistical Analysis

Descriptive analysis was performed. All variables were categorized. Logistic regression models were performed to obtain crude and adjusted odds ratios (OR) and 95% confidence intervals (CI) for the association between explanatory variables and SI. Aiming to control the possible effects of confounders, we examined the associated factors on SI using a stepwise backward procedure where, first, all exposure variables that had *p*-values ≤ 0.20 in the bivariate analysis were entered simultaneously into the model, and then those with *p*-values > 0.05 were progressively withdrawn from the model, until only those with *p*-values < 0.05 were kept in a final model. Statistical analysis was performed using STATA 16 software. All analyses were performed considering the weighting for the complex sample structure, in order to represent the Brazilian population, according to the research sample (SVY command in STATA).

### Ethical Approval

The Brazilian National Survey of Health (PNS-2019) was approved by the National Health Ethics Committee (process 3.529.376) in August 2019. Participation was voluntary and participants signed a consent form. The questionnaire could be completed in whole or in part. The PNS dataset is publicly available on the Brazilian Institute of Geography and Statistics website without information that could identify individuals.

## Results

Of the 27,249 women of reproductive age (15 to 49 years old) in the PNS-2019 data set, we excluded 113 women with uncertain pregnancy status and 26,367 non-pregnant women, leaving a total of 769 pregnant women for the present analysis (Figure 1).

Of the 769 pregnant women included in the present analysis, 36.1% were White, 79.1% lived with a partner, 55.2% lived in the South/Southeast regions of the country, and 44.5% were between 35 and 49 years old. Approximately a quarter of participants (25.8%) had private health insurance, and 40.1% reported a monthly income per capita of more than $1 minimum wage. (Minimum wage in Brazil is the lowest monthly payment for an employee permitted by law. MW = R$ 1.000,00 which was equivalent to $USD250.) These socioeconomic and demographic data are representative of the Brazilian population. Almost three-quarters of participants reported having a very good or good health status (Table 1). The range of the PHQ-9 scores was 0 to 24, the mean score for the PHQ-9 was 4.2 (95% CI: 3.9–4.5), and the mean score for PHQ-9 item 9 was 0.06 (95% CI: 0.03–0.08, range 0 to 3). In relation to AD, 58.6% had a score below 5 (no depression) while 21.1% scored 10 or more (moderate depression). The prevalence of SI was 3.9% (95% CI: 3.0–5.1), including the 33 pregnant women in the sample that reported SI. The proportion of women with SI varied according to the PHQ-9 score: among the 480 non-depressed participants (score <5), the proportion of pregnant women with SI was 0.1% (95% CI: 0.05–0.48); among the 186 pregnant women with mild depression (score 5–9), the proportion of SI was 2.6% (95% CI: 1.5–4.4), and among the 103 pregnant women with moderate depression (score >9), the proportion of women with SI was 17.5% (95% CI: 13.9–21.9).

**Table 1:** Characteristics of suicidal ideation among pregnant women in the Brazilian National Survey of Health, 2019.

**Variable**	***N***	**Prevalence %**	***N***	**Suicidal Ideation %**	
		(95% CI)**		(95% CI)**	***P*** level*
**Age (mean years)**					0.001
15–19	88	10.0 (9.2–10.8)	6	8.6 (5.9–12.3)	
20–34	529	45.5 (42.4–48.5)	20	3.1 (1.7–5.6)	
35–49	152	44.5 (41.5–47.4)	7	3.7 (2.6 – 5.2)	
**Race/color**					0.49
White	226	36.1 (30.2–42.4)	7	4.4 (3.0–6.4)	
Other	543	63.9 (57.5–69.7)	26	3.6 (2.4–5.4)	
**Schooling**					<0.001
0–8 years	244	32.5 (26.2–39.4)	18	8.9 (6.2–12.8)	
9–11	357	41.7 (35.3–48.4)	13	2.3 (1.2–4.1)	
>11	168	25.7 (23.1–28.5)	2	0.2 (0.07–0.8)	
***Monthly income per capita*** ***(minimum wage – Reais)***					0.001
Up to ½	320	31.5 (27.4–35.8)	22	8.2 (6.0–10.9)	
½ to 1	212	28.3 (23.4–33.9)	8	4.0 (2.0–7.7)	
> 1	236	40.1 (35.8–44.6)	3	0.6 (0.1–0.6)	
**Lives with partner**					<0.001
Yes	593	79.1 (76.0- 81.9)	21	2.3 (1.4–3.6)	
No	176	20.9 (18.0- 23.9)	12	10.1 (7.0–14.2)	
**Urban area**					0.86
Yes	580	84.1 (81.0–86.7)	23	3.9 (2.8–5.3)	
No (rural)	189	15.9 (13.2–18.9)	10	4.1 (2.2–7.7)	
***Country region***					0.003
South/Southeast	218	55.2 (49. −60.9)	6	2.1 (1.0–4.2)	
Other	551	44.8 (39.0–50.7)	27	6.4 (5.0–8.1)	
**Private health insurance**					0.03
No	619	74.2 (70.1–77.8)	31	5.0 (3.8–6.6)	
Yes	150	25.8 (22.1–29.9)	2	0.8 (0.1–4.3)	
***Perception of health status***					0.02
Very good/good	601	74.2 (70.7–77.3)	18	2.5 (1.6–3.9)	
Regular	157	24.9 (21.7–28.2)	14	7.5 (5.4–10.2)	
Poor/very poor	11	0.9 (0.4–1.6)	1	23.4 (3.8–70.0)	
***Antenatal depression (PHQ-9 score)***					<0.001
0–4	480	58.6 (53.7–63.3)	4	0.2 (0.07–0.62)	
5–9	186	20.3 (16.9–24.1)	9	2.2 (1.0–4.8)	
10-max	103	21.1 (16.4–26.6)	20	16.0 (11.3–22.1)	

* *P level <0.05 (bold) entered into the multivariate analysis*.

** *Estimated with Stata svy command (to take into account the sample weighting)*.

A higher frequency of SI was found among pregnant women who were 15 to 19 years old (8.6%), with less then 8 years of education (8.9%), among the poorest (8.2%), not living with a partner (10.1%), not living in the South/Southeast regions (6.4%), and without private health insurance (5.0%). Additionally, a higher frequency of SI was seen among participants who reported a poor/very poor health status (23.4%) and moderate depression (16.0%) (Table 2).

**Table 2:** Associations between suicidal ideation, sociodemographic factors, and obstetric risk factors in the Brazilian National Survey of Health, 2019.

**Variables**	**Suicidal**	**Ideation**	
	**Crude**	***P*** **level**	Adjusted *	***P*** **level**
	**OR (95% CI)**		**OR (95% CI)**	
**Age (years)**		0.001		0.013
15/19	1			
20/34	0.34 (0.16–0.70)		0.24 (0.08–0.74)	
35/49	0.41 (0.24–0.70)		0.15 (0.04–0.50)	
**Race/color**		0.49		
White	1			
Other	0.81 (0.44–1.46)			
**Schooling**		<0.001		0.03
0–8 years	1		1	
9–11	0.24 (0.11–0.52)		0.23 (0.61–0.86)	
>11	0.02 (0.007–0.09)		0.15 (0.02–1.17)	
***Monthly income per capita (minimum wage – Reais)***		0.001		0.013
Up to ½	1		1	
½ to 1	0.46 (0.21–1.01)		0.46 (0.15–1.41)	
> 1	0.06 (0.13–0.31)		0.08 (0.01–0.58)	
**Live with partner**		<0.001		
Yes	1			
No	4.73 (2.56–8.72)			
**Urban area**		0.86		
Yes	1			
No (rural)	1.06 (0.51–2.23)			
***Country region***		0.003		<0.001
South/Southeast	1		1	
Other	3.13 (1.48–6.58)		5.52 (2.36–12.9)	
**Private health insurance**		0.03		
No	1			
Yes	0.15 (0.02–0.88)			
***Perception of health status***		0.02		
Very good/good	1			
Regular	3.13 (1.78–5.51)			
Poor/Very poor	11.7 (1.47–94.1)			
***Antenatal depression (PHQ-9 score)***		<0.001		<0.001
0/4	1		1	
5/9	10.8 (2.72: 42.9)		10.5 (2.3–47.9)	
10/max	89.4 (27.8–287.0)		241.3 (58.4–996.7)	

*Adjusted by mother's age, schooling, country region, and antenatal depression.

In the bivariate analysis, the following variables were associated with an increased risk of SI: not having a partner (OR = 4.73; 95% CI: 2.56–8.72), not living in the South/Southeast regions (OR = 3.13; 95% CI: 1.48–6.58), reporting a regular health status (OR = 3.13; 95% CI: 1.78–5.51) or a poor/very poor health status (OR = 11.7; 95% CI: 1.47–94.1), and having mild (OR = 10.8; 95% CI: 2.72–42.9) or moderate AD (OR = 89.4; 95% CI: 27.8–287.0). The following variables were associated with a decreased risk of SI: being 20 to 34 years old (OR = 0.34; 95% CI: 0.16–0.70) or 35 to 49 years old (OR = 0.41; 95% CI: 0.24–0.70), having 9 to 11 years of schooling (OR = 0.24; 95% CI: 0.11–0.52) or > 11 years of schooling (OR = 0.02; 95% CI: 0.007–0.09), and having the achieved the highest family income category (OR = 0.06; 95% CI: 0.13–0.31) (Table 2).

In the adjusted analysis, women had lower odds of SI if they were 20 to 34 years old (adjusted OR = 0.24; 95% CI: 0.08–0.74) or 35 to 49 years old (adjusted OR = 0.15; 95% CI: 0.04–0.50), had 9 to 11 years of schooling (adjusted OR = 0.23, 95% CI: 0.061–0.86), and had achieved the highest family income category (adjusted OR = 0.08; 95% CI: 0.01–0.58). In contrast, pregnant women had higher odds of SI if they did not live in the South/Southeast regions (adjusted OR = 5.52; 95% CI: 2.36–12.9) or had probable mild AD (adjusted OR = 10.5; 95% CI: 2.3-47.9) or probable moderate AD (adjusted OR = 241.3; 95% CI: 58.4–996.7) (Table 2).

## Discussion

The results of our study of a nationally representative sample of women of reproductive age may have public health implications to reduce the burden of SI during pregnancy in LMICs. This study found that SI is prevalent (3.9%) and is strongly associated with depression scores during pregnancy in Brazil. The risk of SI during pregnancy increased in tandem with elevated scores for depression on the PHQ-9. Nevertheless, 0.1% of pregnant women without depression and 2.6% of pregnant women with mild depression also reported SI. Therefore, screening for SI is recommended for pregnant women both with and without depression. Even though there is no commonly used screening tool for SI, a recent study found that screening for multiple psychosocial vulnerabilities can be effective (25). An early identification of SI with screening tools (such as the Edinburgh Postnatal Depression Scale [EPDS] and PHQ-9) should be followed by an appropriate psychiatric assessment. Pregnancy an opportune time to screen for SI as well as depression given the frequent and close contact between women and health providers during antenatal appointments. Further psychological assessments and psychiatric referral would be applied to pregnant women who screened positive for SI. Unfortunately, we believe that factors associated with lower perinatal depression screening by health care providers—such as insufficient training, lack of time during prenatal appointments, competing demands, and difficulties referring the more complex cases (26)—are also obstacles for suicidal ideation screening. Future studies should develop new strategies to assess suicide risk focusing on identifying the risk factors and protective factors for any given individual. At institutional level, public policies should prioritize a more comprehensive assessment of women's psychosocial vulnerabilities in pregnancy, tackling the limited resources of the health system and different levels of barriers to antenatal care (27).

Our findings from Brazil, a LMIC, align with previous studies on the prevalence of SI in high-income countries. For example, a number of investigations in the US have aimed to estimate SI using different methodologies. Gavin et al. (28) studied 2,159 women receiving prenatal care at a university obstetric clinic to identify the prevalence of and factors associated with antenatal SI as measured by the PHQ-9. Overall, 2.7% of the sample reported antenatal SI. Tabb et al. (17) used EPDS and found a prevalence of SI of 4.6% among a sample of 736 low-income US pregnant women. Kim et al. (29) evaluated 22,118 pregnant women from two US hospital of suburban integrated health systems with the EPDS and found an overall prevalence of SI of 3.8% during the perinatal period (4.1% antepartum and 3.5% postpartum). They also also reported that most women with SI were not acutely suicidal according to structured phone interviews performed by trained mental health professionals. All of these estimates are lower than the finding in a study performed in Lima, Peru. Zhong et al. (18) performed a cross-sectional study with 1,517 pregnant women who were assessed for SI using two screening tools: PHQ-9 item 9 and EPDS item 10. They found that 15.8 and 8.8% of participants screened positive for SI with the PHQ-9 and the EPDS, respectively. Of note, both the EPDS and the PHQ-9 are valid tools for assessing depressive symptoms but are not designed to evaluate the full spectrum of suicidality (plans, behaviors, or actions). A limitation of the EPDS is that the scale cannot assume suicidal intent (25).

The SI prevalence found in the present study is similar to both gravid and non-gradid populations in Brazil. For instance, our study is near the results found in a Brazilian prospective study with adults that described the pattern of comorbidity between mental disorders and their association with suicidality (30). In 1982, all hospital deliveries in Pelotas (Southern Brazil) were identified (*n* = 5914) and newborns were followed prospectively. The presence of common mental disorders at the ages of 18–19, 23, and 30 years were evaluated. In 2012–13 (30 years of age), trained psychologists evaluated 3,657 individuals for disorders using the Mini International Neuropsychiatric Interview finding a prevalence of suicidal ideation of 4.9%. Our SI prevalence rate is even higher than the one observerd in a large cross-sectional of Brazilian civil servants (ELSA-Brasil, the Brazilian Health Longitudinal Study, *n* = 15,105). They found that 101 (0.67%) participants presented suicidal thoughts in the 7 days prior to the interview (31). The sample size sociodemographic profile and method of SI assessment may explain the lower rates found in this study in comparions with our results.

Our findings also reaffirm some cross-sectional studies in Brazil also have assessed SI. Huang et al. (16) used the Self-Report Questionnaire 20 (SRQ-20) to evaluate 831 low-income women at 20 to 30 weeks of pregnancy. The prevalence of SI was 6.3%. In comparison with our results, the use of SRQ and the use of a convenience sample from public antenantal care clinics may contribute to a slightly higher prevalence of SI. Da Silva et al. (32) ADDIN EN.CITE evaluated SI during pregnancy among women who had attended public health services between 2006 and 2008 with the question 10 from EPDS. They found that 8.1% of pregnant women reported SI from item 10 of the EPDS (32). Our population-based sample included pregnant women from public and private health services, and lower prevalence of depression and SI is expected among women from the private sector in previous studies. Some of the mechanisms related to lower prevalence of untreated depression captured on screens is from the greater access to mental health care through private insurance for those with higher socioeconomic status. In contrast, Pinheiro et al. (33) evaluated 871 pregnant teenagers receiving prenatal care from the national public health system. They assessed suicidal behavior (and psychiatric disorders) using the Mini International Neuropsychiatric Interview and found a 13.3% prevalence of some form of suicide risk behavior. Possible explantions for higher rates of suicidal behavior in their study are the lower socioeconomic characteristics of the sample and the higher frequency of lifetime history of emotional or physical abuse. The same instrument was used to assess current suicide risk among 225 pregnant women. It found that lifetime suicide attempt rate was 12.55% (34). It is possible that some of the variation in rates in related to the type of scales used.

The large variation in SI seen among studies may be explained by the different methodologies used, including the use of a single item from a depression screening tool and the characteristics of the sample populations. Higher rates of SI are expected among women who report antenatal treatment for neuropsychiatric, epileptic, or mental health conditions (6, 35); who report illicit drug use (36); or who present specific risk factors such as intimate partner violence (15). Finally, pregnant women with lower socioeconomic status experience higher the risk of SI (35). Despite wide variations in past studies, our study confirms the importance of examining a number of psychosocial factors to detect risks for SI.

In the present study we found that younger women experience an increased risk of SI. This is consistent with studies from the US (13), (17) and Brazil (32). Moreover, a UK study evaluated 4,785 women aged 16 to 50 years who died by suicide (2% of whom were in the perinatal period) and found that compared to non-perinatal women, women who died by suicide in the perinatal period were more likely to be younger (37). Younger age also has been associated with SI in studies among the general population. Nock et al. (4) assessed 17 countries to estimate the cross-national lifetime prevalence of SI and reported that age is inversely related to risk for each suicidal behavior, while Borges et al. (38) used data from adults in 21 countries (*N* = 108,705) interviewed with the WHO Composite International Diagnostic Interview to report that younger age is a predictor of suicide attempt. Physical abuse and unplanned pregnancy, which are factors commonly associated with pregnancy during adolescence in Brazil (8, 39), may explain the higher risk of SI among younger women. Additionally, it has been shown that younger women and women who are members of racial/ethnic minority groups are the least likely to receive a diagnosis of depression (40), the most important predictor of SI.

In the present study of a national sample, race/skin color was not associated with SI in the bivariate analysis. This is not an unexpected finding considering that results of published studies from high-income countries have been inconsistent. Some US studies reported a higher risk of suicide attempt among non-Hispanic Whites in comparison with other ethnic groups, while other studies could not find any significant association between race/ethnicity and suicide ideation or attempts (41). In Brazil, a population-based study with 1,295 individuals (18 years or more), living in a Southern city, collected suicidal thoughts using item 9 from the PHQ-9. They did not find a significant association betweem suicidal thoughts (6.6% of participants) and race (42). Because of sample size limitations, this study was unable to examine the many categories by skin color and this could be an area for future research. In the future studies could explore the experience of SI during pregnancy and if skin color or region shapes the experiences of the individuals.

We also found an association between fewer years of schooling and lower family income with SI. These findings have been shown in several studies among pregnant (8, 15, 32, 43) and non-pregnant women in the general population (31, 44, 45). Two studies reported that fewer years of schooling almost tripled the risk of SI during pregnancy (32, 46). Social disadvantage is associated with less access to health care and higher risk of psychiatric conditions, which are related to suicidal behavior. Access to mental health care is one important structural barrier. In South/Southern regions of the country, there is greater availability of mental health care in comparison with other less developed regions. One example is the higher number of psychiatrists in the Southern states (4.55 psychiatrists per 100,000 inhabitants) than in the Northern states (>1 psychiatrist per 100,000 inhabitants). Attaining higher education may help women identify resources to mental health care and improve their outcomes (13).

Our data confirmed results from several studies that have depicted the importance of AD as one of the highest risk factors for SI (8, 13, 15–17, 35, 47). Moreover, the higher the score for depression, the higher the risk for SI. As a matter of fact, other comorbid psychiatric disorders, such as anxiety, panic, substance use, and post-traumatic stress, have been associated with SI (35). Nevertheless, our data also revealed that a small proportion of women without depression or with mild depression reported SI. A similar finding has been reported in different studies. Zhong et al. found that 49% of pregnant women with SI had no depression, with a score of 10 or lower on the PHQ-9. They also found that some participants with a lower PHQ-9 score (>6) still endorsed SI (18). Tabb et al. found that among a sample of low-income pregnant women with SI, 35% did not meet the criteria for elevated depressive symptoms (17). Overall, our results are consistent with data from a study of the general population in Brazil that showed that the prevalence of suicidal thinking is low among mentally healthy individuals and is higher in persons with psychiatric disorders, suggesting that SI is an indirect marker of mental disorders (31).

Screening universally for SI is one approach to detect SI during pregnancy in LMICs. There is a new approach for SI screening recently developed in Sri Lanka. Palfreyman (25) screened 1,000 antenatal women from all trimesters of pregnancy in 2016 using a novel three-part instrument that included the Edinburgh Postnatal Depression Scale, a modified Columbia-Suicide Severity Rating Scale, and Life Circumstances questionnaire. Palfreyman found that one in four women reported a lifetime history of suicidal ideation and/or behaviors (SIB), while SIB during the current pregnancy was reported at 7.4% (25). A higher prevalence found in this study in comparison with our results could be explained by a higher frequency of intimate partner violence and a higher prevalence of depressive symptomology (~one in every three women). Other cultural aspects such as intimate partner violence and child/teen marriage may also play a role.

### Strengths and Limitations

Our study has several strengths. The data in our study were derived from a large population-based research survey that used a complex sampling method that included pregnant women from all socioeconomic statuses. Therefore, generalizability of our findings can be inferred. Moreover, the sampling method allowed the inclusion of populations at high risk for suicidality, such as women with mental disorders. The eventual exclusion of this group of women could contribute to lower prevalence of SI. Moreover, SI screening was assessed among all participants, including those with and those without depression.

Our study also has some limitations. First, the cross-sectional design did not allow us to establish temporal causality. Second, depression was evaluated with the PHQ-9 total score, while self-harming ideation was assessed with a single item from the same instrument. A single question may have different meanings for pregnant women (48). Psychiatric evaluation with the use of structured diagnostic criteria and assessments of other dimensions of suicidality would be very helpful. For example, item 9 of the PHQ-9 does not contain information on intensity or duration of SI, thus preventing a clear understanding of the severity of the suicidality. The PHQ-9 was not originally designed to evaluate risk of suicide (20). Moreover, item 9 from PHQ-9 cannot assess intention, which is a critical element of suicidal behavior and an affirmative answer in this item does not imply an imminent risk of suicide (49). Even though SI reported on the PHQ-9 was found to be a robust predictor of suicide attempts and deaths (50), item 9 covers only non-specific intent of death and self-harm. Nevertheless, the use of the PHQ-9 to measure depressive symptoms instead of depressive disorders, as well as SI, could be justified in population-based studies such as PNS-2019. Third, we also cannot rule out recall bias while using self-reporting to evaluate SI and depression. Therefore, one may expect that the true estimates of SI could be even higher. Finally, we did not assess data about covariates such as interpersonal violence, pregnancy planning, substance abuse, and more specific aspects of partner relationship variables that are commonly associated with SI. These variables may be closed related to pregnant women's suicidal risk and should be assessed in future studies.

## Conclusions

Findings from our study showed that SI is common among 4% of Brazilian pregnant women. We found SI is associated with antenatal depression and a vulnerable psychosocial profile that includes lower socioeconomic status, lower amount of education, and pregnancy during adolescence. SI was also reported among a small group of pregnant women without depression or with mild depression, which is important for clinical approaches to detect SI during pregnancy.

From this national-derived sample, we confirmed findings from clinical samples, although at a lower prevalence, confirming SI is a common problem during pregnancy. In conclusion SI is strongly associated with depression and a vulnerable psychosocial profile in a LMIC and this should serve as a call to action. The correct identification of these risk factors through the use of screening practices may help us to develop preventive strategies for pregnant women at higher risk of SI during pregnancy.

## Data Availability Statement

Publicly available datasets were analyzed in this study. This data can be found here: The Brazilian National Survey of Health (PNS-2019) was approved by the National Health Ethics Committee (process n° 3.529.376) in August 2019. Participation was voluntary and participants signed a consent form. The questionnaire could be completed in whole or in part. The PNS dataset is publicly available on the Brazilian Institute of Geography and Statistics website without information that could identify individuals. Further enquiries can be directed to the corresponding author.

## Ethics Statement

Ethical review and approval was not required for the study on human participants in accordance with the local legislation and institutional requirements. The patients/participants provided their written informed consent to participate in this study.

## Author Contributions

All authors listed have made a substantial, direct, and intellectual contribution to the work and approved it for publication.

## Conflict of Interest

The authors declare that the research was conducted in the absence of any commercial or financial relationships that could be construed as a potential conflict of interest.

## Publisher's Note

All claims expressed in this article are solely those of the authors and do not necessarily represent those of their affiliated organizations, or those of the publisher, the editors and the reviewers. Any product that may be evaluated in this article, or claim that may be made by its manufacturer, is not guaranteed or endorsed by the publisher.
